# Supplementary figures and images for: Evolution of protection after maternal immunization for respiratory syncytial virus in cotton rats
Source: PLoS Pathog. 2021 Dec 23;17(12):e1009856. doi: 10.1371/journal.ppat.1009856 (PMC8741018; doi:10.1371/journal.ppat.1009856)

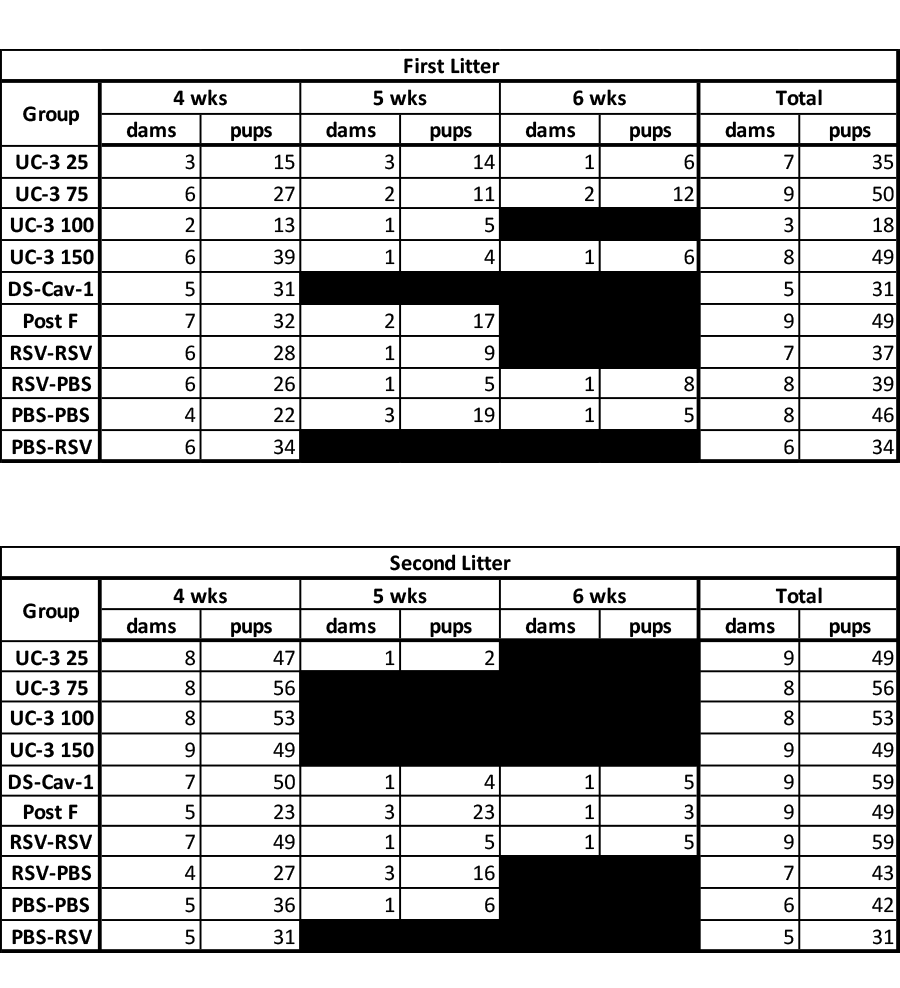

Supplement: S1 Fig — Table shows the numbers of dams and pups from each group giving birth to the first litter of pups (top panel) and second litter of pups (bottom panel) and the times of birth after breeding (day 56 for breed one and day 158 for breed 2). Four weeks indicate pups were delivered four weeks after breeding, while 5 and 6 weeks indicate births at those times after breeding. (TIF) [file ppat.1009856.s001.tif]

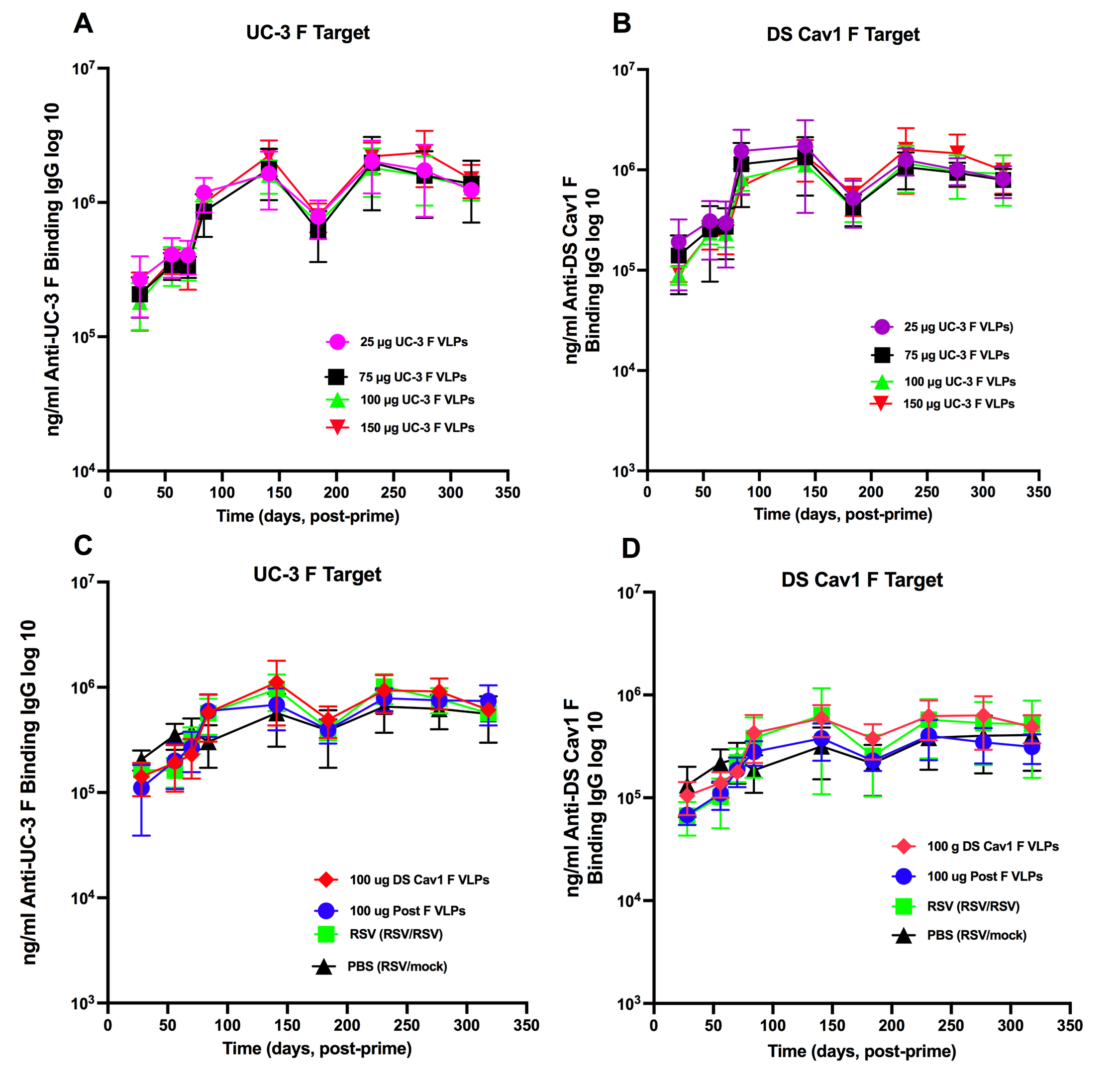

Supplement: S2 Fig — The concentrations of anti-pre-F serum IgG in different groups of dams immunized with VLPs or RSV were assessed by ELISA using soluble UC-3 F (panels A, C) or soluble DS-Cav1 F (panels B, D) as target. Antibody titers in serum samples acquired in different groups of animals at each time after RSV prime and immunized with 25, 75, 100, or 150 μg of 100 μg of UC-3 F are shown in panels A and B. There were no statistically significant differences in titers between these groups of animals. Antibody titers in serum samples acquired in different groups of animals at each time after RSV prime and immunized with 100 μg DS-Cav1 F, or 100 μg post-F VLPs, RSV, or mock immunized are shown in panels C and D. There were no statistically significant differences in titers between these groups of animals. Error bars show mean and standard deviation of three separate determinations. (TIF) [file ppat.1009856.s002.tif]

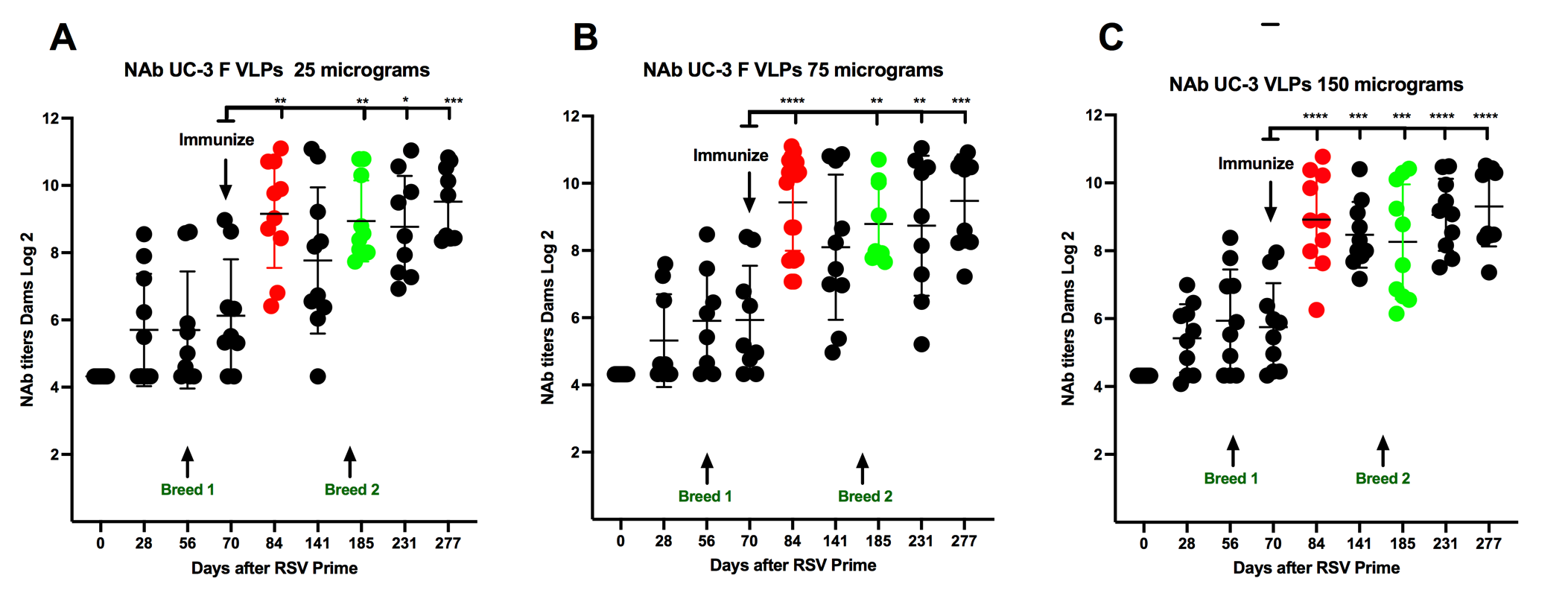

Supplement: S3 Fig — Panels A, B, and C show neutralizing antibody titers of individual animals immunized with 25, 75, or 150 μg of UC-3 F VLPs, respectively. Mean of each group is shown as a black horizontal bar. Red and green dots: data from sera acquired just before delivery of breed 1 or 2, respectively. Significant differences from titers at day 70 were identified by one way ANOVA followed by Sîdák multiple comparisons test; *p<0.05; **p<0.01; ***p<0.001; ****p<0.0001. (TIF) [file ppat.1009856.s003.tif]

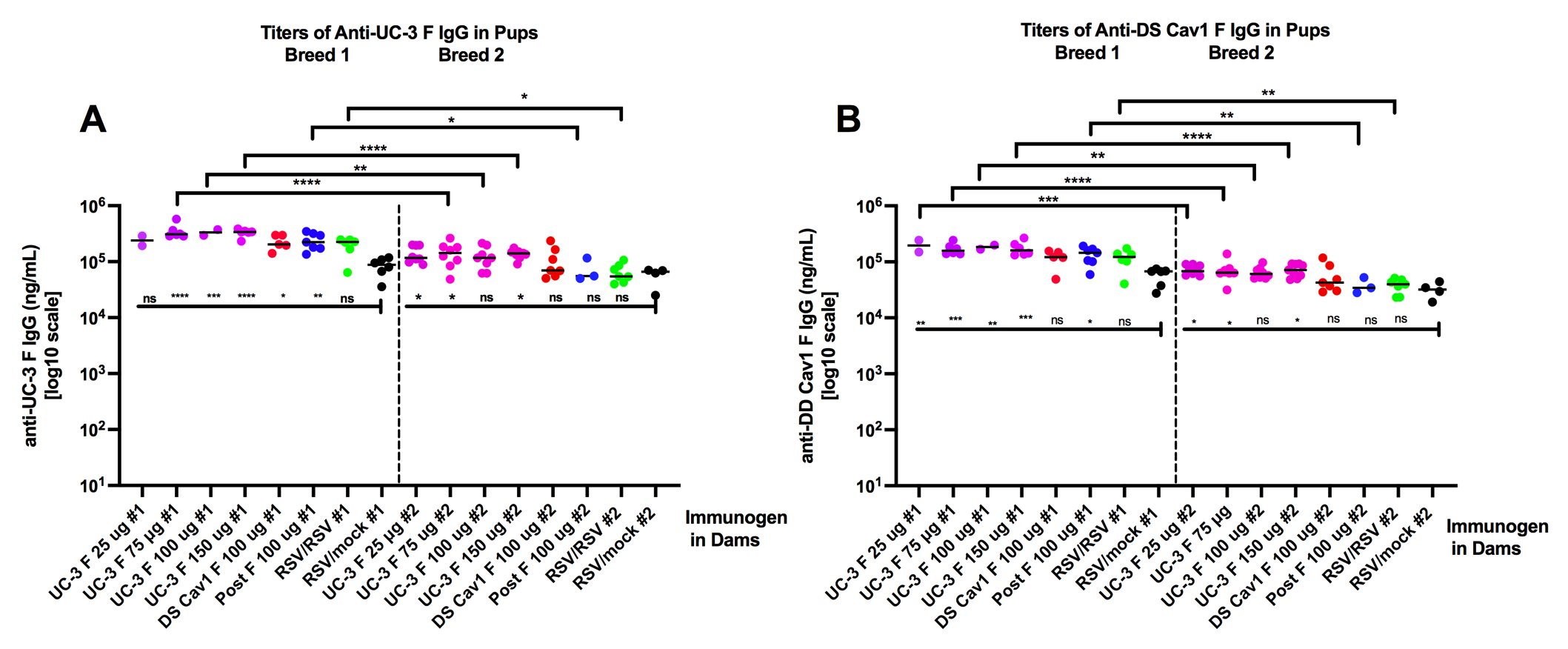

Supplement: S4 Fig — Shown are total anti-pre-F IgG (ng/ml) (shown on a log10 scale) in individual offspring of breed 1 and breed 2 (panel A) dams immunized with 25, 75, 100, and 150 μg of UC-3F VLP, 100 μg of DS-Cav1 VLPs, 100 μg post F VLPs, RSV, or mock immunized. Pre-F IgG titers at four weeks after birth were determined using as target in ELISA soluble UC-3 F (panel A) or DS Cav1 F (panel B). Mean is indicated by horizontal black line. Significance of differences between matching groups in breed 1 vs breed 2 were determined by one way ANOVA followed by Sîdák multiple comparisons test; *p<0.05; **p<0.01; ***p<0.001; ****p<0.0001. *p<0.05; **p<0.01; ***p<0.001; ****p<0.0001. (TIF) [file ppat.1009856.s004.tif]
